# Disruption of Prostate Epithelial Differentiation Pathways and Prostate Cancer Development

**DOI:** 10.3389/fonc.2013.00273

**Published:** 2013-10-31

**Authors:** Sander B. Frank, Cindy K. Miranti

**Affiliations:** ^1^Laboratory of Integrin Signaling and Tumorigenesis, Van Andel Research Institute, Grand Rapids, MI, USA; ^2^Genetics Graduate Program, Michigan State University, East Lansing, MI, USA

**Keywords:** prostate cancer, differentiation, Myc, Pten, notch, p38MAPK

## Abstract

One of the foremost problems in the prostate cancer (PCa) field is the inability to distinguish aggressive from indolent disease, which leads to difficult prognoses and thousands of unnecessary surgeries. This limitation stems from the fact that the mechanisms of tumorigenesis in the prostate are poorly understood. Some genetic alterations are commonly reported in prostate tumors, including upregulation of Myc, fusion of Ets genes to androgen-regulated promoters, and loss of Pten. However, the specific roles of these aberrations in tumor initiation and progression are poorly understood. Likewise, the cell of origin for PCa remains controversial and may be linked to the aggressive potential of the tumor. One important clue is that prostate tumors co-express basal and luminal protein markers that are restricted to their distinct cell types in normal tissue. Prostate epithelium contains layer-specific stem cells as well as rare bipotent cells, which can differentiate into basal or luminal cells. We hypothesize that the primary oncogenic cell of origin is a transient-differentiating bipotent cell. Such a cell must maintain tight temporal and spatial control of differentiation pathways, thus increasing its susceptibility for oncogenic disruption. In support of this hypothesis, many of the pathways known to be involved in prostate differentiation can be linked to genes commonly altered in PCa. In this article, we review what is known about important differentiation pathways (Myc, p38MAPK, Notch, PI3K/Pten) in the prostate and how their misregulation could lead to oncogenesis. Better understanding of normal differentiation will offer new insights into tumor initiation and may help explain the functional significance of common genetic alterations seen in PCa. Additionally, this understanding could lead to new methods for classifying prostate tumors based on their differentiation status and may aid in identifying more aggressive tumors.

## Introduction

Prostate cancer (PCa) is the most common non-skin cancer and second leading cause of cancer deaths in American men (1). Treatment for locally confined PCa is highly effective and typically involves radiation therapy or removal of the prostate gland. Such treatment, however, is not without considerable financial cost and a potentially negative impact on quality of life for the patient. Gleason grade, as determined by biopsy, is a moderate predictor of tumor aggressiveness, but even small, low grade tumors can become aggressive and metastasize (2). Researchers are currently working to understand and create better criteria to identify which primary tumors will be indolent and which will be aggressive (3). The ability to clearly identify which tumors are most likely to metastasize would potentially save thousands of men from unnecessary surgery when they are more likely to ultimately die of other causes (4). At the same time, the ability to accurately and reliably identify aggressive tumors would aid physicians and patients in deciding how to administer treatment. When detected too late or left untreated, PCa becomes metastatic and the primary therapeutic option becomes androgen deprivation. Despite initial effectiveness, androgen deprivation therapy invariably leads to the emergence of castration resistant disease which is incurable and accounts for the vast majority of the nearly 30,000 PCa deaths each year (1).

Whether contemplating cancer or a mechanical watch, it is impossible to fix something without understanding how it broke. Through years of work, PCa researchers have identified what is broken but are still working to understand how it broke. Although we know which oncogenes and tumor suppressors are most frequently altered in PCa, we do not understand how they drive tumor initiation (i.e., oncogenesis) (5). Without a thorough understanding of oncogenesis researchers will continue to struggle for a comprehensive understanding of PCa. The PCa field is currently stuck with a host of critical but unanswered questions: why are these particular genes mutated so frequently in PCa? How do the alterations drive oncogenesis? Does the timing and order of these mutations dictate tumor aggressiveness?

There are a few oncogenes and tumor suppressors that are mutated across a wide range of cancers, such as Ras and p53 (6, 7). However, most cancers contain a distinctive set of “driver mutations,” thus revealing multiple oncogenic routes that are highly dependent on not only the specific pathways disrupted but also the cell of origin (8). Many of the genes commonly altered in PCa are also involved in normal epithelial differentiation. With these observations in hand, we hypothesize that prostate oncogenesis arises as a defect in epithelial differentiation. Unfortunately, research is lacking in understanding the detailed signaling mechanisms of normal prostate differentiation. In this review we will describe key genes that are altered in PCa and what is known about their roles in epithelial differentiation. Additionally, we will propose some hypothetical models for how oncogenic alterations of these important pathways could potentially drive a normal trans-differentiating prostate epithelial cell to become tumorigenic.

## Prostate Background

**Figure 1:**
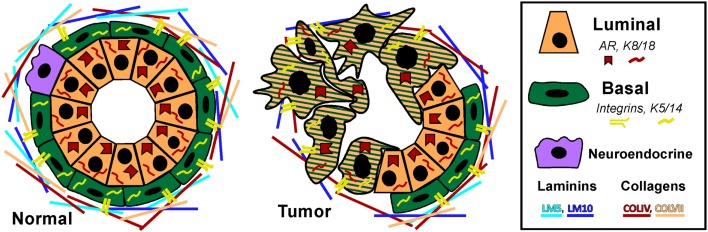
**Prostate epithelial gland structure**. The normal prostate epithelium is composed of a bilayer of basal and luminal cells and a few rare neuroendocrine cells. The epithelium is separated from the underlying stroma by a basement membrane containing laminins (LM5, LM10) and collagens (COLIV, COLVII). Basal cells express integrins that specifically interact with the basement membrane, namely α6β4, α3β1, and α2β1 as well as basal keratins K5 and K14. Luminal cells do not express integrins, but do express AR and keratins K8 and K18. A prominent characteristic of prostate tumors is the loss of basal cells and a loss of LM5 and COLIV. Correspondingly, the integrins that interact with these matrices, α3β1 and α6β4 are lost via loss of the α3 and β4 subunits, leaving integrin α6β1 which prefers the LM10 matrix. Similarly, tumor cells often co-express basal and luminal keratins, such as K5 and K8.

Prostate adenocarcinoma arises from the epithelia of the gland. Prostate epithelium is organized in a bilayer of basal and luminal cells along with a few rare embedded neuroendocrine cells (Figure 1). The epithelium is surrounded by a laminin (LM5, LM10) and collagen (COLIV, COLVII) matrix and fibromuscular stromal cells which transmit signals to regulate the epithelium (9). The prostate epithelium contains layer-specific markers, with the basal layer characterized by p63, basal keratins (K5, K14), and integrins (α6β4, α3β1) among others. The luminal layer contains markers such as TMPRSS2, luminal keratins (K8, K18), and androgen receptor (AR). Prostate tumors are characterized by a loss of basal cells and reduced matrix diversity (i.e., loss of LM5 and COLIV) (Figure 1). Moreover, tumor cells generally express a luminal phenotype driven by AR. However, tumors also express basal integrins, especially α6β1 which is an abnormal pairing that drives PCa growth and survival (10, 11). Similarly, tumor cells often co-express basal and luminal keratins, such as K5 and K8 (12–14). Other basal markers reportedly expressed in tumor cells include Bcl2, EGFR, and Met (15–20). The co-expression of a subset of luminal and basal markers supports the hypothesis that prostate tumors arise from the disruption of differentiation pathways which normally restrict basal and luminal marker expression to their respective cell types. However, the cell of origin, i.e., the cell that is the oncogenic target that gives rise to the tumor, has not been clearly identified in PCa.

The struggle to define a clear cell of origin is complicated by the fact that the mechanism of prostate epithelial differentiation is not well understood. In the adult prostate, luminal cells are regularly shed and replaced by cells from the basal layer through differentiation (21). A simplistic view of this observation is that a basal progenitor or stem cell gives rise to the both basal and luminal populations through a transient-differentiation or amplification process (12, 22–24). However, findings from mouse models paint a more complicated picture of basal, luminal, and bipotent progenitors. Ousset et al. utilized cell lineage tracing to clearly demonstrate the existence of layer-specific epithelial progenitor cells in the developing mouse prostate (25). Wang et al. demonstrated that a luminal progenitor, marked by expression of Nkx3.1, resists luminal regression induced by castration and repopulates the majority of the mouse prostate during regeneration with androgen (26). On the other hand, using tissue recombination and renal capsule implants, Leong et al. showed that a single prostate stem cell is able to produce both epithelial layers (27). The Witte group also identified basal stem-like cells in the mouse prostate that produce both basal and luminal cells (28–31). Other researchers also identified a small population of bipotent progenitor cells that give rise to both basal and luminal cells (25, 26). These rare bipotent cells are marked by their co-expression of basal and luminal keratins (K5/K8) and are also found in the developing human prostate (25, 32). Thus, the mouse studies support the idea there are at least three different prostate epithelial progenitor populations, but which ones initiate PCa still remains unresolved.

Several studies demonstrate either basal or luminal progenitors can be the initiating cancer cell. The Witte group demonstrated that oncogenic disruption in the basal cell population drives tumor formation in mice (33–35). On the other hand, two groups reported that both basal and luminal epithelial cells can give rise to tumors upon knockout of PTEN (14, 36). Thus, mouse studies suggest distinct stem cell populations may be responsible for tumor initiation and seemingly disfavor the transient amplification theory. However, introduction of genetic mutations early in development and puberty in mice does not reflect the normal situation in humans were oncogenesis occurs in a fully differentiated gland. Moreover, the signals and cell types that regulate gland maintenance vs. development may be different. Transgenic mouse models rely on Cre recombination via commonly used “layer-specific” promoters, such as K5 or Nkx3.1. Thus, these studies still leave open the possibility that it is not a pure basal or luminal cell that becomes oncogenic, but rather a bipotent or transient-differentiating cell expressing multiple layer markers. Better understanding of prostate differentiation will be required to validate the transient-differentiating hypothesis of oncogenesis.

While studies in the mouse are highly informative, translation of these findings to understanding the human organ is complicated due to a lack of models for studying human oncogenesis. The mouse model is useful for genetic manipulations, but it is not without limitations (37, 38). For example, mice are not prone to develop spontaneous PCa like humans. Secondly, although the mouse and human prostate have similar cell types, the structure is different; the mouse prostate is lobular while the human is compact and consists of zones (39, 40). Additionally, there are far fewer basal cells in the mouse prostate and some luminal cells directly contact the basement membrane, unlike in humans where there is a continuous layer of basal cells. Based on these important differences, there is reason to consider that signaling mechanisms for differentiation in human and mouse epithelial cells may be different. As an alternative to transgenic mouse models, some researchers are using human prostate cells and xenografts in mice to study prostate development and differentiation. The Cunha group found that human basal cells can be induced to form a basal and luminal bilayer when combined with rat urogenital sinus mesenchyme and implanted in the mouse renal capsule (41). The Witte group developed a similar method to isolate and genetically modify epithelial progenitor cells from human prostates (42). The isolated progenitor cells were infected with virus to allow manipulation of desired oncogenes/tumor suppressors, and then the cells were implanted into mice along with stroma. Using this approach, they found that the induction of Akt and Erg in human basal progenitors is sufficient to induce prostate intraepithelial neoplasia (PIN), a PCa precursor lesion, when xenografted into mice (33). Other groups are inducing the differentiation of primary basal cells *in vitro*, including our group which has developed a reliable *in vitro* differentiation model that recapitulates many aspects seen *in vivo* (17, 43–45). These reports demonstrate that human basal cells can be induced to differentiate into luminal cells *in vitro*, thus providing a model to study epithelial differentiation in a controlled setting using human cells. The ability to manipulate cells *in vitro* during differentiation and then implant them into mice provides a useful approach to study how manipulation of trans-differentiating human prostate epithelial cells can become tumorigenic.

Based on the slowly building knowledge of normal prostate differentiation, as well as findings from other epithelial tissues, it is becoming apparent that many of the pathways involved in normal epithelial differentiation are misregulated in PCa. In this review, we will describe some of these key pathways that are involved in both differentiation and cancer, with the goal of illuminating how prostate oncogenesis in humans may arise from a disruption of normal differentiation. Furthermore, aggressive tumors are pathologically characterized by a less differentiated phenotype, and the aggressiveness of the tumor may be tied to its cell of origin (14, 36). Better understanding of prostate differentiation pathways will help us understand how the normal cellular process goes awry in cancer. This understanding may one day lead to classification of prostate tumors based on the state of their altered differentiation pathways.

## Common Genetic Alterations in Prostate Cancer

While there are still no widely accepted subcategories of PCa, there are well established genetic alterations associated with the disease. Fundamentally, prostate tumors rely on AR signaling. While AR mutations are rare in primary tumors, castration resistant tumors utilize a variety of genetic alterations to upregulate AR signaling (46–48). Beyond the AR alterations in advanced tumors, three of the most common genetic alterations in PCa are: overexpression of Myc, loss of the tumor suppressor Pten, and fusion of Ets genes with upstream AR regulated promoter sequences (e.g., TMPRSS2-Erg) (49, 50).

The Myc gene is commonly amplified in PCa (Table 1) and protein levels correlate with poor prognosis (51). Myc is a well-studied oncogene that drives the expression of thousands of targets, including genes required for cell growth and cell cycle progression. Myc overexpression in the mouse prostate is sufficient to drive adenocarcinoma but not metastasis (52). The importance of Myc in PCa is well established, though not entirely understood, and will be discussed in further detail in the next section.

**Table 1:** **Myc overexpression in PCa**.

	Tumor type	Method	Citation
**% 8q GAIN^a^**			
38	Primary + LN met	SNP, qPCR	Liu et al. (108)
27	Primary + LN met	CGH	Lapointe et al. (53)
72	CRPC	CGH	Nupponen et al. (54)
**% 8q24 GAIN^a^**			
9	Primary (LG)	FISH	Gurel et al. (50, 55)
28	Primary (HG)	FISH	Gurel et al. (50, 55)
**% Myc GAIN^a^**			
77	CRPC	FISH	Nupponen et al. (54)
21	Primary	DNA array	Edwards et al. (56)
63	CRPC	DNA array	Edwards et al. (56)
**Myc IHC SCORE**			
2.6	Normal	IHC	Gurel et al. (50, 55)
8.6	LG-PIN	IHC	Gurel et al. (50, 55)
25.8	HG-PIN	IHC	Gurel et al. (50, 55)
27.1	Primary	IHC	Gurel et al. (50, 55)
14.9	Met	IHC	Gurel et al. (50, 55)

*^a^ Percentage of tumors displaying the change*.

*LN, lymph node; CRPC, castration-resistant PCa; LG, low grade; HG, high grade; Met, metastasis*.

Another prevalent aberration in PCa is loss of the tumor suppressor Pten (Table 2), a negative regulator of the PI3K pathway. At least one copy of the Pten locus is lost in up to 65% of prostate tumors and complete loss of Pten protein is seen in ∼60% of late stage tumors (57–62) Loss of one copy of Pten greatly increases PCa progression in the TRAMP mouse model and Pten dosage has a marked impact on tumor latency and progression (63, 64). Moreover, complete loss of PTEN in the mouse prostate is sufficient to drive adenocarcinoma (65, 66). The role of Pten in PCa and differentiation will be discussed in further detail in a later section.

**Table 2:** **Pten loss in PCa**.

	Tumor type	Method	Citation
**% Gen Del^a^ (1×)**			
39	PIN	FISH	Yoshimoto et al. (67)
20	Primary	FISH, CGH	Verhagen et al. (61)
30	Primary	Sequencing	Barbieri et al. (59)
31	Primary	FISH	Yoshimoto et al. (62)
36	Primary	FISH	Lotan et al. (60)
65	Primary	Sequencing, PCR	Gray et al. (57)
**% Gen Del^a^ (2×)**			
5	Primary	FISH	Yoshimoto et al. (67)
6	Primary	FISH	Yoshimoto et al. (62)
22	Primary + CRPC	FISH, PCR	Verhagen et al. (61)
20	Met	FISH	Yoshimoto et al. (68)
**% MUTATION^a^**			
4	Primary	Sequencing	Barbieri et al. (59)
8	Primary	Sequencing	Verhagen et al. (61)
14	Primary	Sequencing	Gray et al. (57)
**% PROTEIN LOSS^a^**			
12	PIN	IHC	Lotan et al. (60)
40	Primary	IHC	Lotan et al. (60)
40	Primary	IHC	Verhagen et al. (61)
60	Met	IHC	Lotan et al. (60)

*^a^ Percentage of tumors displaying the change*.

*Del (1×), deletion of one chromosome; (2×), deletion of two chromosomes; Gen Del, gene deletion; CRPC, castration-resistant PCa; Met, metastasis*.

Activation of the Ets pathway is also a common occurrence in PCa (Table 3), most frequently through the fusion of the oncogene Erg downstream of the androgen-regulated promoter of TMPRSS2 (69, 70). Specific genetic rearrangements that drive tumor progression are relatively rare in solid cancers, but the TMPRSS2-ERG fusion is a notable exception and is observed in about 50% of prostate tumors (53, 62, 71–74). The identification of additional fusions of AR-driven promoters to other Ets members (as well as other targets) strongly suggests this type of rearrangement is a major driver of PCa (75–80). This has important implications about the role of AR in PCa development and may explain the dependency on AR for tumorigenesis. In the normal secretory epithelium, AR is primarily required for maintaining secretory functions and is not intrinsically required for survival or proliferation of the secretory epithelium (81, 82). In fact, AR is inhibitory to cell proliferation in normal cells (83, 84). But an opposite response is triggered in tumor cells, where both proliferation and survival depends on AR. The trigger is unknown, but prostate-specific oncogenes driven by AR are likely to be part of the answer.

**Table 3:** **TMPRSS2/Erg fusions in PCa**.

% With fusion^a^	Tumor type	Method	Citation
13	PIN	qPCR	Furusato et al. (73)
20	PIN	FISH	Perner et al. (74)
45	Primary	FISH	Yoshimoto et al. (62)
50	Primary	FISH	Perner et al. (74)
67	Primary	qPCR	Furusato et al. (73)
30	Met	FISH	Perner et al. (74)

*^a^ Percentage of tumors displaying the change*.

*Met, metastasis*.

Thus, the contribution of AR-driven Ets activation and the mechanisms that drive tumor initiation and tumor progression are in need of much further investigation. The Ets family of transcription factors can potentially regulate a wide range of cellular processes, including development, differentiation, invasion, and proliferation (85). Sun et al. reported that the TMPRSS2-Erg fusion activates Myc and prevents terminal prostate epithelial differentiation in the VCaP PCa line (77). Additionally, Yu et al. reported that Erg and AR-binding sites have considerable overlap and Erg functions in part by disrupting AR binding to its target genes in VCaP cells (76). Moreover, Yu et al. found that Erg activates EZH2, which is part of the polycomb repression complex and in turn down regulates an AR-driven differentiation program. The authors propose that TMPRSS2-ERG is likely to be an early mutational event that drives selection of cells with hyper activated or mutated AR to overcome the antagonistic effects of Erg activation on AR (76). Conversely, Chen et al. using transgenic mice and reported that Erg activation aids AR signaling by increasing AR binding to target genes, though only in the context of Pten loss (86). The Chen et al. group suggest that an explanation for the difference in their findings from those of Yu et al. is that the latter did their studies in VCaP cells, which retain Pten expression. If the Chen et al. finding is to be believed, then Ets activation may be a later event that must follow Pten loss. There are multiple mouse models of Erg overexpression, but only some of them produce PIN and none develop adenocarcinoma (75, 87–89). In the most aggressive model, overexpression of the N-terminal truncated Erg fusion product in luminal cells (via a modified probasin promoter) produces PIN in about 40% of mice but still fails to drive adenocarcinoma (75). The combination of Erg overexpression with single-copy loss of Pten drives progression to adenocarcinoma but does not result in metastasis (87, 88). These findings from mouse models further support the idea that Ets activation is a later event in PCa progression and must follow Pten loss. More research on Ets and its specific function in PCa tumorigenesis and/or progression are required to fully understand the significance of this common mutation.

As will be discussed in this review, pathways that are frequently altered in PCa (Myc, Pten, Erg) can be tied to normal prostate differentiation. Likewise, key epithelial differentiation pathways (p38MAPK, Notch) are also misregulated in human and mouse models of PCa. We propose the hypothesis that the ties between oncogenesis and differentiation are evidence that PCa arises from a transiently differentiating prostate epithelial cell. Moreover, while these differentiation pathways may not be direct drivers of PCa, we believe they are critical for oncogenesis via potential misregulation of Myc and PTEN during aberrant differentiation. In this review, we will discuss what is known about Myc, p38MAPK, Notch, and Pten and their roles in both cancer and differentiation with the goal of providing new insight into understanding PCa oncogenesis.

## Myc

### Myc background

The general importance of Myc in PCa is well established, but it is less clear precisely how Myc drives tumor initiation and progression (51). In addition to its oncogenic role, Myc is also crucial for promoting epithelial differentiation (90, 91). Knowledge about normal prostate differentiation is limited, and much of it is based on mouse studies. More detailed investigations into the role of Myc in prostate differentiation may help us understand how its misregulation leads to PCa.

There are three genes in the Myc family: c-Myc, N-Myc, and L-Myc. c-Myc (Myc) is the best studied and most relevant in PCa. Myc is a basic helix-loop-helix transcription factor that typically functions as a heterodimer with a cofactor from the Max or Miz families (92). Transcriptional regulation by Myc is mediated through recruitment or activation of basal transcription machinery, promoting RNA Polymerase II elongation, or through recruitment of chromatin modifying enzymes (93, 94). The Myc/Max heterodimer is usually a transcriptional activator complex that competes with Mad/Max dimers for binding at E-box sites, the classic regulatory element recognized by Myc complexes. Myc also represses genes by binding with Sp1 or Miz1, which together repress transcription by blocking p300 (95, 96). Alternately, Myc can repress targets post-transcriptionally via activation of miRNAs (97, 98).

Myc is downstream of many pathways and is tightly regulated at the mRNA and protein levels (95, 99). Myc mRNA and protein have short half-lives and higher activity is usually associated with lower stability (100). Myc potentially regulates thousands of genes, with one estimate predicting as much as 15% of the genome (101, 102). While there are thousands of potential targets for Myc, its functional role in cellular processes is highly dependent on the level of expression, duration of activation, and expression of its cofactors.

### Myc in prostate cancer

The vast majority of prostate tumors overexpress Myc (Table 1), which correlates with poor prognosis (51, 103, 104). While Myc mRNA is elevated in as many as 80% of prostate tumors, there is less certainty about Myc protein levels (51). The De Marzo group published a study showing that Myc protein expression is very low in normal prostate epithelium but higher and more nuclear localized in PIN and prostate tumors (55). The most common mechanism of Myc overexpression is through amplification of the gene locus, usually through gain of 8q. The narrower Myc region 8q24 is more selectively amplified in late metastatic tumors (50, 53, 105–108). However, early prostate tumors also overexpress Myc but rarely have Myc amplifications, suggesting other mechanisms driving Myc overexpression which are less well understood (51). Myc amplification is specifically observed in castration resistant tumors (54, 56). Bernard et al. demonstrated that Myc overexpression in the hormone-sensitive LNCaP line confers resistance to androgen deprivation or AR knockdown (109). Conversely, AR knockdown decreases Myc expression, indicating Myc is downstream of AR. In another study, the ability of AR to upregulate Myc was ligand independent (110). Alternatively, Myc reportedly upregulates AR, suggesting there may be feedback mechanisms between the two proteins (111, 112).

Another potential mechanism for Myc upregulation is via β-catenin, the downstream target of Wnt signaling. Constitutive β-catenin is sufficient to upregulate Myc and induce prostate tumor formation in a mouse model (113). Furthermore, the APC gene (an antagonist of β-catenin) is often silenced by hypermethylation in at least 50% of human prostate tumors (114, 115). However, the specific role of APC and β-catenin in human PCa is still unclear and it is unknown if the potential oncogenic activity is due to Myc upregulation. As will be discussed later, some groups report increased Notch signaling in prostate tumors, which may also drive transcription of Myc (116, 117).

Myc overexpression in the mouse prostate with a weak promoter drives low grade PIN but not adenocarcinoma (118). Using stronger variants of the probasin promoter to regulate Myc overexpression in luminal cells, researchers were able to drive progression to adenocarcinoma though not metastasis (52). In this model, when Myc is driven by the endogenous probasin promoter (Lo-Myc) mice take much longer to develop tumors than those with a stronger promoter (Hi-Myc) (52). Finally, mice with knockout of Mxi1 (a Myc antagonist) show prostate dysplasia but do not develop adenocarcinoma (119). All together, these models demonstrate that Myc can drive PCa in the mouse, and the level of Myc expression is related to the aggressiveness of carcinoma that develops.

Myc has many potential oncogenic and tumor-promoting targets. One group of genes known to be regulated by Myc is cell cycle regulators, such as E2F members, cyclins, and cyclin-dependent kinases (120). Additionally, Myc can regulate cell growth by upregulating tRNAs and rRNAs (120). Other important targets of Myc include stem cell genes, such as hTert and EZH2 (51, 76, 120). Myc is one of the four original genes whose overexpression was initially used to create pluripotent stem cells, along with Oct4, Sox2, and Klf4 (121). Although overexpression of Myc was later found not to be necessary for stem cell induction, Myc activity is required for embryonic stem cell self-renewal (122–125). Another key point regarding Myc is that the level and timing of its expression is critical for deciding what function the protein will play, for example deciding whether Myc drives proliferation or stem cell maintenance (120). However, which of these targets is critical for PCa development and progression is not clear. In summary, Myc amplification is common in late metastatic tumors and can act as a driver in mouse models but specific mechanisms of Myc regulation and downstream targets are poorly understood.

### Myc in prostate differentiation

Beyond its multifaceted role in cancer, Myc is also important for differentiation. A shift from Myc/Max to Mad/Max binding is associated with terminal differentiation (126, 127). Transient expression of Myc aids induced pluripotent stem cell transformation while sustained expression stimulates down regulation of integrin α6 and drives differentiation of embryonic stem cells (128). In keratinocyte differentiation, Myc protein is expressed in the basal layer and decreases during differentiation of the suprabasal layers (129, 130). On the other hand, knockdown of Myc prevents *in vitro* keratinocyte proliferation while transient overexpression induces premature terminal differentiation (131–133). Overall a short, high spike in Myc appears to be required for proliferation, while a more moderate and extended increase in Myc is characteristic for differentiation (91). The role of Myc specifically in prostate differentiation has not been well investigated. Preliminary data from our lab indicate Myc follows a pattern of moderate increase over a brief period that is required during prostate epithelial differentiation in our *in vitro* model using primary human basal cells (unpublished data).

One mechanism by which Myc triggers differentiation is through its control of a cell adhesion program. About 40% of the genes downregulated upon Myc activation in mouse skin are involved in cell adhesion and cytoskeleton, including integrins α6, β1, and β4 (132). Integrin loss as the cells from the basal layer rise into the upper layers triggers keratinocyte differentiation (134). This adhesion profile is largely regulated via Miz1, given that a Myc mutant unable to bind Miz1 loses the ability to suppress integrin α6 and β1 transcription (132).

Another mechanism by which Myc may regulate differentiation is via interactions with chromatin remodeling proteins (94, 135, 136). Chromatin modifications are often associated with cell programing, such as patterns for stem or terminally differentiated cells (136, 137). Pellakuru et al. published a study looking at Myc and H3K27me3 in prostate differentiation and cancer (136). H3K27me3 is a marker of polycomb activity, which induces heterochromatin and gene repression. The group reported that basal prostate cells have lower levels of H3K27me3 than luminal cells as determined by immunostaining with human tissue sections (136). Furthermore, using a tissue micro array they also found that cases of human PIN show decreased H3K27me3 compared to normal luminal cells. Levels of H3K27me3 are also decreased in prostate tumors from Hi-Myc mice. Additionally, they showed that Myc knockdown in the PC3 and LNCaP PCa lines leads to an increase in H3K27me3 (136). The authors were unable to provide a mechanism for how Myc controls H3K27me3, but they previously reported that Myc upregulates EZH2, which is the catalytic member of the polycomb complex and is often overexpressed in PCa (136, 138). However, EZH2 overexpression does not correlate with higher H3K27me3 levels in this study, which led the authors to propose that regulation of EZH2 activation may be a separate event (136). Seemingly answering the idea proposed by Pellakuru et al. a later study reported that EZH2, upon phosphorylation at Ser21, plays a non-polycomb role in castration resistant PCa acting as an AR coactivator (139).

### Myc conclusion

Myc amplifications are very common in advanced prostate tumors but Myc is also upregulated in early tumors through currently unknown mechanisms (51). Normal upregulation of Myc is required for proliferation and differentiation, and it is the level and timing of Myc expression that largely determines which of those decisions the cell will make (91). The upregulation of Myc seen in PCa may explain how tumors arise from a transient amplifying or differentiating prostate cell which requires a temporary upregulation of Myc expression. However, additional oncogenic events are required to prevent terminal differentiation and death due to the oncogenic stress of sustained Myc activation (95). As typically happens with other cancers, loss of p53 can relieve apoptotic stress; however, p53 loss is a rare event in primary prostate tumors and is usually only seen in a small subset of metastatic tumors (140). Thus, other yet to be identified mechanisms must be involved in PCa development. Abnormal Myc expression and its role in regulating a cell adhesion program may also help explain why prostate tumors show a large general loss in integrin and matrix expression, except for the retention of the tumor-promoting integrin α6β1 pairing (11, 132, 141). Additionally, prolonged Myc activation in a transient-differentiating cell may drive changes in chromatin structure, as evidenced by the fact that basal and intermediate prostate cells show low levels of heterochromatin markings compared to tumors (136). There is accumulating evidence to suggest that Myc contributes to an altered differentiation program in PCa, but more studies are required to work out particular mechanisms.

## p38MAPK

### p38MAPK background

The three classic branches of mitogen-activated protein kinase (MAPK) signaling are p38MAPK (p38), Erk, and Jnk. MAPK signaling involves kinase cascades that control a wide range of functions in the cell including proliferation, stress response, and differentiation (142). The MAPK pathways can regulate gene expression through a variety of mechanisms at the RNA and protein levels. Erk signaling is most classically associated with growth factor signaling, while Jnk and p38 are commonly associated with stress responses to insults such as inflammation and radiation (143). p38 and Jnk have specific direct upstream kinases: MKK3/6 activate p38 and MKK4/7 activate Jnk (though MKK4 can potentially activate p38 in some cases) (142). However, p38 and Jnk share some common activating kinases further upstream, such as Ask1 and Tak1 (143). This upstream convergence makes identifying the contribution of each pathway difficult. In epithelial differentiation, upstream p38 activation is via the receptor tyrosine kinase FGFR2, specifically the FGFR2b (FGFR2IIIb) isoforms, by KGF (FGF7) or FGF10 ligands (43, 144, 145). MAPKs are also negatively regulated by a host of MAPK phosphatases, which are dual-specificity protein phosphatases that inactivate MAPK members (146). While the MAPK pathways share some overlapping features, p38 has a distinctive role in epithelial differentiation (142).

There are four isoforms of p38: MAPK14 (p38α), MAPK11 (p38β), MAPK12 (p38γ), and MAPK13 (p38δ). They share about 60% gene homology and have some compensatory ability, though they also have differential target preferences (142). p38α is the most prevalent and ubiquitously expressed, while p38β is moderately expressed in many tissues and p38γ/δ are more tissue specific. The p38 kinases can signal through many different effectors, including other kinases, phosphatases, transcription factors, and mRNA binding proteins (142). Due to this range of potential targets, p38 can regulate gene expression at the transcriptional, post-transcriptional, and post-translational levels.

### p38MAPK in prostate cancer

There are a handful of studies which investigated p38 signaling in PCa (Table 4). Unfortunately, most studies only interrogate p38α (generally referred to as p38). In the TRAMP mouse model of PCa, Uzgare et al. reported that p38 is highly activated in PIN lesions and more well-differentiated tumors but is absent in late stage and metastatic tumors (147). However, most other studies report that p38 activation correlates with PCa progression and treatment with a p38 inhibitor in a rat PCa model led to decreased angiogenesis and reduced tumor formation (148). Utilizing 25 primary prostate tumors and a combination of immunoblotting, ELISA, and IHC, Royuela et al. reported that phospho-p38 (p-p38) is upregulated in prostate tumors (149). Based on immunoblot analysis, tumors showed ∼50% higher expression of p-p38 than normal prostate. Furthermore, about 17% of normal prostate epithelium stains positive for p-p38 while nearly 90% of the tumor samples were positive (149). Additionally, a report by Lotan et al. demonstrated that MKK4 and MKK6 proteins are minimally expressed in normal prostate luminal cells, moderately expressed in basal cells, and highly upregulated in PIN lesions from human and mouse (TRAMP model) (150). However, this study did not look specifically at the active (phosphorylated) MKK proteins and also found that total MKK4/6 levels are not statistically different in low vs. high grade tumors (150). Ricote et al. looked at upstream (MKK6) and downstream (ATF-2, Elk-1) p38 targets in PCa progression (151). They reported that MKK6 is not detected in normal prostate samples, but it appears upregulated in PCa. Also, they detect p-ATF-2 and p-Elk-1 protein in normal basal cells but expression of both is higher in PCa (∼2.5- and ∼3-fold, respectively). ATF-2 and Elk-1 are also potential Jnk targets, but the authors did not detect any Jnk in the PCa samples so they attributed all of the ATF and Elk activation to p38 (151). Together, these reports suggest upregulated p38 activity in PCa progression, at least in part due to upregulation of the upstream activating kinases, such as MKK6.

**Table 4:** **p38 Signaling pathway in PCa**.

	Tumor type	Method	Citation
**p-p398α PROTEIN^a^**			
1.0	Normal	WB	Royuela et al. (149)
1.2	BPH	WB	Royuela et al. (149)
1.5	Primary	WB	Royuela et al. (149)
1.0	Normal	IHC	Royuela et al. (149)
3.5	BPH	IHC	Royuela et al. (149)
5.0	Primary	IHC	Royuela et al. (149)
**MKK4 PROTEIN^b^**			
0.3	Normal	IHC	Lotan et al. (150)
2.4	HG-PIN	IHC	Lotan et al. (150)
0.7	Normal	IHC	Lotan et al. (150)
1.9	Primary	IHC	Lotan et al. (150)
**MKK6 PROTEIN^c^**			
1.0	Normal	IHC	Lotan et al. (150)
2.6	HG-PIN	IHC	Lotan et al. (150)
0.9	Normal	IHC	Lotan et al. (150)
2.0	Primary	IHC	Lotan et al. (150)
29.0	BPH	WB	Ricote et al. (151, 152)
70.0	Primary	WB	Ricote et al. (151, 152)
**p-Elk-1 PROTEIN^c^**			
13	Normal	WB	Ricote et al. (151, 152)
47	BPH	WB	Ricote et al. (151, 152)
34	Primary	WB	Ricote et al. (151, 152)
**p-ATF-2 PROTEIN^c^**			
5	Normal	WB	Ricote et al. (151, 152)
14	BPH	WB	Ricote et al. (151, 152)
22	Primary	WB	Ricote et al. (151, 152)
**% MKP-1 PROTEIN^d^**			
100	PIN	ISH	Loda et al. (153)
94	Primary (LG)	ISH	Loda et al. (153)
28	Primary (HG)	ISH	Loda et al. (153)
0	Met	ISH	Loda et al. (153)
100	BPH	IHC	Rauhala et al. (154)
12	Primary	IHC	Rauhala et al. (154)
3	CRPC	IHC	Rauhala et al. (154)

*^a^ Relative to normal samples*.

*^b^ IHC score, + 1, + 2, + 3*.

*^c^ Average intensity*.

*^d^ Percentage of tumors staining in medium to high range*.

*CRPC, castration-resistant PCa; LG, low grade; HG, high grade; Met, metastasis*.

MKP-1 (DUSP1) is a nuclear MAPK phosphatase that antagonizes Jnk and p38α/β activation (146). Several reports indicate MKP-1 is overexpressed in early prostate tumors but is downregulated in high grade and castration resistant tumors, as well as a portion of PIN lesions (153–157). MKP-1 can be activated by p38 in a negative feedback mechanism, so it is possible that down regulation of MKP-1 may be a necessary precursor to p38 upregulation in more advanced PCa tumors (158).

IL-6, a key regulator of inflammation, is also linked with PCa and p38 signaling (159–162). Ueda et al. reported that IL-6 activates transcription of AR targets in a p38-dependant manner in LNCaP cells (159). Ricote et al. reported that TNFα, a cytokine and known activator of MAPK stress response, induces apoptosis in LNCaP cells but not PC3. Moreover, TNFα activates p38 in LNCaP cells, and p38 inhibition increases apoptosis (152). Building on that finding, Gan et al. reported that LNCaP cells can be sensitized to docetaxel by blocking p38, which prevents p53 activation and apoptosis (163). Moreover, this was not observed with PC3 or DU145 cells, which do not have functional p53. These findings were supported by a second group which further investigated the role of p53 in docetaxel resistance in the same cell lines (164). Thus, over-activation of p38 is likely to trigger an apoptotic response without additional pathway alterations to compensate, which may include p53 loss in a subset of late PCa tumors but also likely involves other unknown mechanisms.

Though the FGFR2b receptor is crucial for differentiation, there are reports suggesting that growth factors such as EGFR and IGF1R can also activate p38 (165–167). Prostate tumors often show downregulation of FGFR2b and KGF (FGF7) and upregulation of other FGFs and FGFRs which drive proliferative (168). Overexpression of FGF10 in mouse prostate stromal cells causes adenocarcinoma when combined with normal mouse prostate epithelia and implanted in the mouse renal capsule (169). Furthermore, the degree of tumor progression correlated with the amount of FGF10-expressing stroma implanted, suggesting a dose-dependent function of FGF10 (169). Additionally, the FGF10 driven tumors are more resistant to androgen deprivation. This group also found that blocking FGFR1 activation in the epithelium with a dominant-negative mutant rescued oncogenic transformation, while dominant-negative FGFR2 only moderately reduced invasion (169). Moreover, another study reported that FGFR1 activation in prostate epithelium could drive PCa in the mouse (170). Whether the ability of FGF10 or FGFR1 to drive tumorigenesis is dependent on p38 was not determined. Thus, it is likely that the oncogenic potential of FGF10 is not through FGFR2b. Together, these findings support the idea that FGFR2b, which is a potential tumor suppressor in the prostate, inhibits tumor formation by driving differentiation (via p38) instead of proliferation (via other MAPKs or PI3K) (171). Moreover, alternate mechanisms of upstream p38 activation may contribute to PCa progression.

### p38MAPK in prostate differentiation

p38 Promotes differentiation in a range of tissues including intestine, lung, bone, and cornea (172–175). Most research has focused on p38α and much less is known about the expression of specific p38 isoforms in the prostate. Our lab detected mRNA for all four isoforms in human prostate epithelia, but protein only for p38α and p38δ (unpublished data); the latter is often associated with endocrine glands but is also expressed in other epithelial cells such as keratinocytes (176). p38α knockout in mice is embryonic lethal, while p38γ or p38δ knockout results in apparently normal mice (177, 178). Despite the lack of an obvious phenotype, Schindler et al. that found p38δ^−/−^ mice have normal skin but are resistant to skin tumor formation (179). While p38δ may have overlapping functions with p38α, there is evidence that it also has some unique functions that are not well defined (179, 180).

How p38 regulates epithelial differentiation is not well understood. In muscle differentiation, p38α/β (and possibly p38γ) activate MyoD and Mef2 transcription factors and the SWI-SNF chromatin remodeling complex, both of which are required for muscle differentiation (181). Other roles for p38 include inhibiting proliferation, which is a necessary prerequisite for differentiation (182–184). More specifically, p38 activity represses Erk and Jnk, which is reported to be a cellular switch from proliferation to differentiation (180, 183, 185). While p38 is essential in a range of differentiation models, investigation of its role in prostate differentiation is lacking, as is an understanding of the contribution of specific isoforms. However, p38 and its role in other differentiation models may serve as a good starting point for further investigation within the prostate.

Unlike other growth factors, KGF (FGF7) is an epithelial-specific differentiation factor that is typically secreted by surrounding stroma (186–188). KGF and FGF10 bind the same receptor, FGFR2b, and share many overlapping functions including upstream activation of p38 signaling (43, 144, 145). KGF or FGF10 is sufficient to drive prostate differentiation *in vitro* (17, 43). In mouse knockout models, FGF10 and FGFR2 are both required for proper development of the prostate (189, 190). Additionally, FGF10 overexpression can drive tumor formation as previously discussed, suggesting that the dosage of FGF10 is very important for proper prostate homeostasis (169). Thus, FGFR2b signaling through p38 is likely a critical step for prostate differentiation and aberrant expression of FGF ligands and receptors promotes PCa.

### p38MAPK conclusion

p38 activation correlates with PCa progression in many reports (149) (Table 4). Activation of p38 in PCa may be due to a combination of upregulated upstream kinases (MKK3/6) and downregulated MAPK phosphatases (150, 155–157). MKP-1 (DUSP1), which targets p38α/β, is often downregulated in late stage PCa tumors, which suggests p38δ may act as an early oncogenic activator while p38α is a late contributor. However, p38δ appears to play a tumor suppressive role in mouse skin and it would be useful to investigate p38δ in prostate tumors to see if its loss correlates with p38α overexpression (179). Alternatively, the role of p38 in PCa may be dictated by its activating receptor. This idea is supported by observations in PCa showing that more aggressive prostate tumors shift from expression of FGFR2b to FGFR2c, which would prevent differentiation and induce proliferative signals (171). Before the loss of FGFR2b, over activated p38 may drive basal cells to differentiate prematurely, which may partially explain the lack of basal cells in PCa tumors and the mixture of basal and luminal markers in cancer cells.

## Notch

### Notch background

Notch is well known for its role in cell fate decisions, such as stem cell renewal, development, and differentiation (191). There are four Notch transmembrane receptors in rodents and mammals, Notch1–4, that are activated by transmembrane ligands on adjacent cells (192). In mammals, there are five classic ligands from two families: Jagged (Jag1/2) and Delta-like (Dll1/3/4). Recent work demonstrates that Notch signaling can also be activated by a variety of non-canonical proteins, such as Dlk1/2, LRP1, and TPS2 (193). The Notch receptor protein undergoes an initial cleavage event upon emergence from the ER and is then transported to the cell membrane. Upon ligand binding a second cleavage is initiated by ADAM10 and a final cleavage by the γ-secretase complex. The cleaved C-terminal receptor fragment, known as the Notch Intracellular Domain (NICD), translocates to the nucleus where it binds the repressive CSL protein [also known as RBPJκ, CBF1, Su(H), or Lag1] (192). The NICD/CSL complex recruits co-activators such as Mastermind-like (MAML1/2/3) and p300 which trigger a switch from repression to activation of the classic Notch target genes of the hairy and enhancer of split (Hes) family: Hes1-7 and Hey1/2/L (194). After activating transcription, the NICD fragment is quickly degraded and the Hes/Hey factors typically function in negative feedback loops by repressing their own transcription, thus critically controlling the temporal regulation of Notch. Notch also directly activates transcription of other targets, including p21/CDKN1A and Myc (195). While there is some overlap, the four different NICDs have some differential preferences for ligands and downstream targets, though these details are not thoroughly resolved (192, 196, 197). Adding to the complexity, Notch and CSL are reported to have some independent functions and do not always require each other for signaling (198–201). There are additional mechanisms of Notch regulation, such as endosomal and proteosomal turnover of the receptor as well as post-translational modifications of the ligands and receptors; these regulatory mechanisms will not be discussed in depth here but can be found in a variety of reviews (192, 194, 202).

### Notch in prostate cancer

The Notch pathway is misregulated in many cancers, though the type of misregulation is tumor and cell-type specific (203, 204). The most studied model is T-cell Acute Lymphoblastic Leukemia, where Notch signaling is over activated in the majority of tumors (205–207). In other cancers, such as cutaneous and lung squamous cell carcinoma, Notch is understood to be a tumor suppressor (208). Notch1 loss drives skin cancer progression in mice in a non-cell autonomous matter due to loss of barrier cell function, which triggers an immune and growth cytokine response within the tumor microenvironment (209).

Within the PCa field, there are conflicting reports about whether the Notch pathway is tumor suppressive or oncogenic (191, 210, 211). Supporting the case for Notch as a tumor suppressor, Belandia and colleagues reported that Hey1 and HeyL are excluded from the nucleus upon the transition from benign to carcinoma in human prostate samples (212, 213) (Table 5). Furthermore, the same group showed that Hey1 and HeyL bind to AR and potentially function as AR co-repressors in the LNCaP line (213). Other studies similarly found a decrease in Notch1 and Hey1 protein in human PCa tumors compared to normal tissue (214, 215).

**Table 5:** **Notch signaling in PCa**.

	Tumor type	Method	Citation
**% Hey1 NUCLEAR^a^**			
93	BPH	IHC	Belandia et al. (212)
20	Primary	IHC	Belandia et al. (212)
**% HeyL NUCLEAR^a^**			
100	BPH	IHC	Lavery et al. (213)
22	Primary	IHC	Lavery et al. (213)
**Jag1 PROTEIN^b^**			
1.0	BPH	IHC	Zhu et al. (116)
2.1	HG-PIN	IHC	Zhu et al. (116)
0.9	Primary (LG)	IHC	Zhu et al. (116)
3.0	Primary (HG)	IHC	Zhu et al. (116)
3.8	Met	IHC	Zhu et al. (116)
1.0	BPH	IHC	Santagata et al. (216)
1.2	Primary	IHC	Santagata et al. (216)
1.6	Met	IHC	Santagata et al. (216)
**NOTCH1 PROTEIN^b^**			
1.0	BPH	IHC	Zhu et al. (116)
1.4	HG-PIN	IHC	Zhu et al. (116)
1.0	Primary (LG)	IHC	Zhu et al. (116)
2.2	Primary (HG)	IHC	Zhu et al. (116)
4.4	Met	IHC	Zhu et al. (116)
**NICD1 PROTEIN^b^**			
3.6	Normal – basal	IHC	Whelan et al. (215)
2.7	Normal – luminal	IHC	Whelan et al. (215)
1.1	Primary	IHC	Whelan et al. (215)
**% NOTCH3 PROTEIN^c^**			
23	Primary (GG <3)	IHC	Danza et al. (217)
95	Primary (GG >4)	IHC	Danza et al. (217)

*^a^ Percentage of tumors with nuclear staining*.

*^b^ Relative to benign samples*.

*^c^ Percentage of tumors with high staining*.

*LG, low grade; HG, high grade; Met, metastasis; GG, Gleason grade*.

Conversely, several reports demonstrate increased levels of Jag1 and Notch1 protein in high grade PCa tumors, implicating Notch as an oncogene (116, 117, 216, 218) (Table 5). Bin Hafeez et al. observe higher Notch1 protein staining in more aggressive prostate tumors: 64% positive staining for Gleason grade 4 tumors, 30% for high grade PIN, and 5% for normal tissue. Moreover, knockdown of Notch1 in PC3 cells decreases metastatic gene expression and decreases invasion *in vitro* (117). Furthermore, knockdown of CSL, which ablates downstream Notch activity, leads to decreased proliferation in PC3 PCa cells (219). Other groups reported that siRNA knockdown of Notch1 or Jag1 in PC3 cells decreases PC3 growth and colony formation, in part due to an increase in cell death (220, 221).

While most research has focused on Notch1 or overall Notch activity, there are also a few papers reporting a specific role for Notch3 in PCa. Using prostate tumors with known patient outcome, Long et al. found Notch3 mRNA levels positively correlate with PCa recurrence (222). Moreover, of a 12-gene mRNA panel, Notch3 has the second highest prognostic ability for recurrence (222). Ross et al. reported that Notch3, Jag2, and Presenilin1 (a catalytic subunit of the γ-secretase complex) mRNA transcripts are upregulated in high grade prostate tumors (157). Notch is also implicated in PCa via a role in hypoxia. Exposure of LNCaP, PC3, and DU145 cell lines to prolonged hypoxia leads to down regulation of Notch1/2 mRNA and protein but has no effect on Notch3 (223). A follow up report found that hypoxia also induces changes in cholesterol and lipid rafts in the cell membrane, which increases colocalization of Notch3 and γ-secretase, leading to increased NICD3 expression (217). The study also measured Notch3 protein levels in PCa tumor sections and found Notch3 protein levels correlate positively with Gleason grade, thus supporting the Notch3 mRNA correlation reported by Long et al. (217, 222).

### Notch in prostate differentiation

When it comes to cell fate decisions, Notch signaling is critical across most cell types. The Notch pathway has been studied in the prostate to some extent, but knowledge about specific mechanisms and signaling pathways is lacking. Many studies have been conducted in the mouse which, as discussed earlier, has some significant structural differences from the human. Treatment of rat prostates *ex vivo* with a γ-secretase inhibitor prevents lumen formation and treatment with the inhibitor *in vivo* prevents prostate regeneration following castration (220). A similar finding was reported for mouse prostates treated with γ-secretase inhibitors (224). As for receptor-specific studies, Notch1 is the most studied. Wang et al. used an interesting model where they made a transgenic mouse with a lethality gene (bacterial nitroreductase) under control of the Notch1 promoter, which would only be lethal in the presence of an inducing chemical (220). They took early developing mouse prostates and grew them *ex vivo* with or without the inducer and found that ablation of Notch1-expressing cells prevents proper organoid development and differentiation (220). In a follow up study, they utilized γ-secretase inhibitors and an interferon-inducible Notch1 mouse (Mx-Cre/Notch1^flox^) to study the effect of Notch1 loss on prostate development (214). They found that induced Notch1 knockout (in all cells of the prostate including the stroma) leads to increased proliferation and prostatic hyperplasia as well as co-expression of basal and luminal keratins (214). Moreover, Wu et al. utilized transgenic mice to investigate Notch in prostate development, reporting that Nkx3.1-Cre driven CSL knockout leads to decreased proliferation and differentiation defects in the prostate (225). Conversely, Notch1 constitutive activation (via PB-Cre or Nkx3.1-Cre driven NICD1) in the mouse prostate causes increased proliferation and hyperplasia (225). Both of these studies suggest that Notch signaling is required for proper differentiation, while Notch1 specifically appears to be crucial for maintenance of a proper and distinct basal layer. Notch1 also regulates p63, which is a classic basal cell marker in the prostate and a regulator of cell adhesion, including integrins (226–228). Therefore it is intriguing to consider that Notch signaling during prostate differentiation may need to strike a balance between downregulating adhesion through p63 while Notch1 must also maintain homeostatic basal cells. The balance between multiple Notch receptors and downstream targets may in fact be crucial for regulating the decision to stay basal or differentiate.

Studies on other Notch receptors in the mouse prostate are limited; though there are some studies in other tissues. For instance, Notch3 knockout mice develop normally (229). The NICD3 appears to be a weaker activator of downstream signaling than NICD1 and may actually antagonize NICD1 by competing for CSL (230). Dang et al. reported that constitutive Notch3 expression (via NICD3) in mouse lung epithelium prevents terminal differentiation and causes metaplasia and reduced epithelial branching (231). In esophageal differentiation, Notch1 activates Notch3, which in turn activates Hes5 and drives differentiation (232). In skin differentiation, Notch1/2/3 have all been detected in the interfollicular epidermis, but there is a shift in ligands from Jag2 in the basal layer to Jag1 in the upper layers (233). Moreover, in the hair follicle Notch1 is expressed primarily in the bottom of the niche, while the upper regions mainly express Notch2 or Notch3 (233). Such a mechanism where there is a shift in Notch receptor, ligand, and/or downstream targets during prostate differentiation is conceivable but has not yet been investigated.

Due to the structural differences in the mouse vs. the human prostate and the highly context-specific nature of the Notch pathway, further studies are needed to understand the role of Notch in human prostate tissue. For example, in mouse skin Notch1 and Notch2 are mainly expressed in the upper layers; however, in human skin Notch1 is expressed in all layers and Notch2 is mainly restricted to the basal layer (234). There have not been any reports clearly and uniformly demonstrating which components of the Notch pathway are expressed at the protein level in the normal human prostate (191). However, Wang et al. reported mRNA for all four receptors and most Hes/Hey members are expressed in human prostate samples, but only Notch1 and Hey1 levels are altered in PCa tumors (214). Notch1 is the most well-studied receptor in the prostate and it is found predominantly in basal cells of both mouse and human prostates (218, 235). Research on the other receptors is much less abundant. Recently, one study investigated Notch2 and Notch3 expression in PCa progression. They found low levels of Notch3 staining in normal prostate sections and decreased Notch2 expression with increasing tumor grade but did not report whether they detected Notch2 in normal prostate tissue (217). In a mouse model, Notch2 and Dlk1 protein were detected in developing mouse prostate stroma but not the epithelium (224). Understanding the specific role for each Notch receptor in prostate will require further investigation.

### Notch conclusion

While it is established that the Notch pathway is important for normal prostate differentiation, and it appears to be deregulated in PCa, specific details are still ambiguous. The Notch field is still working to define clear roles for the various ligands, receptors, and downstream targets. Moreover, any resolved mechanism is likely to be tissue and cell specific, as is common with this pathway (234). The lack of an NICD3-specific antibody makes identifying its contribution difficult to assess. Additionally, the transient nature of NICD activation and turnover makes it difficult to detect endogenous NICD via histological techniques or immunoprecipitation. Additionally, many studies rely on γ-secretase inhibitors, which fail to distinguish the function of specific Notch receptors. The same applies to studies that use knockdown of CSL to ablate Notch signaling. The limitations of γ-secretase inhibitors or CSL knockdown are apparent in a study from Yong et al. which saw differential effects depending on which technique they used (219). The explanations for such discrepancy are likely due to γ-secretase involvement in other Notch-independent functions and CSL-independent Notch signaling (200, 201). Specific knockdown of individual Notch components is more arduous but allows for a better understanding of the pathway.

There is conflicting data about whether Notch is acting as a tumor suppressor or an oncogene in PCa. While the role of Notch1 in normal prostate differentiation has been investigated, specific mechanisms remain elusive. One interesting idea is that Notch1 is known to transcriptionally upregulate Myc, which may explain how Notch signaling can function as an oncogene (236). The link between Notch-mediated repression of p63 could also explain why prostate tumors show loss of p63 (227). Notch as a tumor suppressor may be mediated through Hey2/L expression, which then acts as an AR co-repressor. Based on the complexity of Notch signaling, it is feasible that its role as an oncogene or tumor suppressor is dependent on which receptors and downstream transcription factors are being activated. For example, if Myc is the primary target, the pathway may be oncogenic; however, if HeyL is the primary target, it may be tumor suppressive. Due to the complexity of the Notch pathway, merely looking at a small selection of the ligands, receptors, or downstream factors may only be providing a small piece of the overall puzzle. As is seen with skin, temporal changes in Notch ligand expression are characteristic of differentiation (233). Perhaps the status of the Notch pathway in prostate tumors can be indicative of the cell of origin or be used to grade relative differentiation status. More thorough investigations of Notch signaling may help clarify its function in differentiation and resolve some of the conflicting findings about its role in oncogenesis and tumor progression.

## PI3K/Pten

### PI3K/Pten background

The PI3K pathway regulates multiple processes in the cell including metabolism, proliferation, and survival (237). Multiple aspects of the PI3K pathway are frequently misregulated in many cancers (238, 239). PI3K is a lipid kinase that targets phosphoinositides, catalyzing the conversion of phosphatidylinositol 4,5-biphosphate (PIP2) to phosphatidylinositol 3,4,5-triphosphate (PIP3). There are three classes of PI3K genes that all target PIP2 but each class varies by structure, regulation, and tissue specificity. PI3K class I members are implicated in cancer and are the most ubiquitously expressed. The class I PI3K family contains four members (PIK3CA, PIK3CB, PIK3CG, and PIK3CD) that code for the catalytic p110 subunit, while three other genes (PI3KR1-3) code for at least five isoforms of the p85 regulatory subunit (238). The p110α and p85α subunits are implicated most often in cancer (238, 239). Activation of a variety of receptors induces recruitment of the regulatory subunit, p85α, which brings the catalytic subunit, p110α, to the cell membrane to catalyze the conversion of PIP2 to PIP3. PIP3 recruits Akt which is then phosphorylated by Pdk1 and the mTORC2 complex. Activated Akt phosphorylates and activates the mTORC1 complex, which in turn regulates a range of targets such as S6K and 4EBP1, both of which are required for protein synthesis (240). Akt also phosphorylates other proteins commonly implicated in cancer, including GSK3β, BAD, MDM2, and p27 (CDKN1B) (238).

While mTORC1 activation by Akt can stimulate a negative feedback mechanism, the primary negative regulator of the PI3K pathway is Pten, a lipid phosphatase that converts PIP3 back to PIP2 and thus antagonizes Akt activity (58). However, Pten has functions beyond its role in regulating PI3K/Akt activity. First, the phosphatase region of Pten is related to other tyrosine phosphatase proteins and dual-specificity protein dephosphatases (58, 241) and as such can also dephosphorylate proteins like focal adhesion kinase (FAK) and cAMP response element binding protein (242, 243). Secondly, Pten has lipid-independent functions within the nucleus such as DNA repair via activation of RAD51 (58, 244, 245). Pten also interacts with p53 to arrest cell growth upon oxidative damage (246–248). Complete loss of Pten leads to major defects in DNA repair (245, 249). Finally, Pten also regulates cell polarity (250, 251).

Pten loss (via mutation, transcriptional repression, or deletion) is common in many cancers, including PCa (58, 252). Because of the ability for PTEN to regulate PI3K/Akt-dependent and independent pathways, it has the capacity to impact many aspects of cellular function, including DNA damage response, FAK activation, and cell polarity. Identifying the most important targets for the establishment, maintenance, or progression of prostate tumors is essential for a full understanding of PCa.

### PI3K/Pten in prostate cancer

Loss of Pten expression in PCa was first recognized in the late 1990s and genetic loss of at least one allele occurs in as many as 65% of metastatic prostate tumors (57, 253, 254) (Table 2). Homozygous deletion of Pten occurs in <10% of primary tumors but occurs in about 20–50% of metastatic, castration resistant tumors (60, 61, 68). Genomic loss of Pten correlates with late stage PCa tumors and poor prognosis (60, 67, 255–257). Alterations in other members of the PI3K pathway are less common than Pten loss. Akt mutations are very rare and <5% of primary PCa tumors have mutations in p110α (PIK3CA), though 1–15% have amplification of the gene locus (5, 59, 258). Mutations in p85α (PIK3R1) are also very rare, though genomic deletions of its locus on 5q are more frequent and expression is downregulated in about 20% in primary tumors and 60% of metastases (5). Multiple studies looking at Pten protein expression by IHC found that while Pten deletions are readily apparent by protein loss, about 30–40% of tumors with loss of Pten protein do not show genomic loss of the Pten gene (60, 61, 259). While Pten deletions are quite common, mutations in the gene itself are rare in primary prostate tumors (5, 260, 261). Pten silencing by methylation is fairly common in other cancers but is not widely reported in PCa (262). Inactivation of Pten via alternate mechanisms, such as miRNA or competing-endogenous RNA, was recently reported, indicating this mechanism might be active in PCa (263–267).

Constitutive Akt expression in the mouse prostate, driven by the probasin promoter, leads to PIN but not tumor invasion, as is seen with total Pten knockout (65, 66, 268). These findings, in addition to the fact that Pten deletion is more common than other PI3K/Akt alterations, suggests that Pten contributes to prostate oncogenesis through multiple mechanisms beyond activation of PI3K/Akt signaling. Pten loss correlates with the TMPRSS2-Erg fusion in human tumor samples (5, 259, 269, 270). Furthermore, Erg activation combined with single-copy Pten loss accelerates PCa progression from PIN to adenocarcinoma in mice (87, 88, 271). In the TRAMP model, Pten haploinsufficiency in the prostate gives rise to larger tumors and decreased survival rates compared to the normal TRAMP mice (63). In humans, it is still not clear if the prognostic association of single-copy Pten loss is due to Pten haploinsufficiency or loss of heterozygosity, i.e., inactivation of the second allele via an alternate mechanism (60).

### PI3K/Pten in prostate differentiation

While loss of Pten in PCa is well established, knowledge about how its loss contributes to tumor development and its role in normal prostate differentiation is very limited (14, 17, 272, 273). Nonetheless, PI3K and Pten are known regulators of epithelial differentiation in a variety of other models (274–278). The Rivard group reported that PI3K is required for E-cadherin assembly at tight junctions, which in turn activates Akt, downregulates Erk, and drives differentiation and survival (279–281). Calautti et al. utilized mouse primary keratinocytes to demonstrate that PI3K signaling is required for the initiation of differentiation and survival of differentiated suprabasal cells (282). Moreover, they found that PI3K signaling and differentiation require EGFR and E-cadherin engagement (282). Supporting these findings, luminal prostate cells also require E-cadherin and PI3K signaling for survival (17). However, the differentiated luminal cells, unlike the basal cells, do not express or require EGFR (17, 283). Thus, PI3K/Akt signaling is crucial for the emergence and survival of differentiated epithelia.

Most notable is the observation that Pten’s role in differentiation extends beyond regulating PI3K/Akt signaling. In a mammary *ex vivo* organoid model, Pten knockout causes disorganization of the epithelial bilayer (284). Moreover, Akt/mTORC1 signaling is necessary but not sufficient for proper differentiation in this model (284). Groszer et al. found that loss of Pten drives premature hematopoietic stem cell differentiation and increases the number of leukemia initiating cells (285–287). Using a kidney epithelium 3D culture model, the Mostov group found that Pten and the balance between PIP2 vs. PIP3 levels is necessary for establishing basal/apical polarity and lumen formation (250, 288). Upon siRNA knock down of Pten, the cells lost actin and PIP2 enrichment at the apical membrane which is required for recruitment of Annexin2 and subsequently Cdc42. Cdc42 then recruits Par6, which together function in a complex with Par3 and aPKC, which is known to regulate cell polarity (251, 289, 290). Pten may be targeted to the apical membrane via its association with tight junctions, though the precise mechanism is unknown. It was also reported that integrin β1 and laminin were required for establishment of basal polarity in the same kidney epithelium model, thus demonstrating that Pten and integrins likely cooperate to establish cell polarity (291). The Rivard group, using an intestine epithelium model, reported that Pten knockdown with shRNA leads to a loss of cell polarity and disruption of adherence junction formation (292). They followed up that study by creating an intestine-specific Pten^−/−^ mouse model and found defects in differentiation *in vivo* (293). Thus, PI3K signaling and Pten both appear to be required for epithelial differentiation. PI3K may be primarily involved in survival of the differentiating cells while Pten appears to be important for establishing cell polarity which is required for organization of epithelial structure. Therefore, epithelial differentiation likely requires a delicate balance between Pten and PI3K activity; for example, Pten may be higher in early differentiation to set up polarity and lower in later differentiation to permit PI3K signaling for survival. Alternately, Pten levels may remain constant throughout differentiation with upregulation of PI3K signaling by other means in the new luminal cell.

### PI3K/Pten conclusion

Downregulation of Pten aids tumor growth (238). However, complete loss of Pten in normal cells can lead to cell arrest or apoptosis (245). In PCa, the initial loss of one copy of Pten may aid growth of the tumor, but other oncogenic events may be required for survival upon loss of heterozygosity. These additional events could include upregulation of other survival pathways, or in later stage tumors, loss of p53. Our lab previously reported that PCa cells utilize the combination of AR and α6β1 to activate a survival pathway independent of PI3K (11). Basal prostate cells do not require PI3K for survival, but luminal cells do (17, 283). Thus, it is conceivable that a transiently differentiating cell may not yet depend on PI3K for survival but already be activating AR and beginning to display the luminal phenotype. This hypothetical cell would combine the tumorigenic benefit of PI3K independent survival with AR activation, which in this context now becomes oncogenic. Additionally, loss of Pten likely disrupts polarity leading to disorganization of the bilayer, and altered homeostatic signaling between the stroma and epithelium, ultimately disrupting differentiation, which may be a prerequisite for tumorigenesis.

## Crosstalk and Oncogenesis Models

### Overview

As covered in this review, Myc, p38, Notch, and PI3K/Pten all play key roles in regulating differentiation and are all misregulated during oncogenesis. Myc upregulation and Pten loss are well established drivers of prostate oncogenesis. However, p38 and Notch have not yet been widely labeled as oncogenic drivers, though we believe that their misregulation in a transiently differentiating bipotent cell can contribute to misregulation of Myc and Pten and help drive oncogenesis. All these pathways are capable of crosstalking with each other, which may help explain their interconnected functions within the prostate. One of the great challenges facing PCa researchers is to understand prostate oncogenesis, which will allow better understanding of how to diagnose and treat PCa. Though there is much more investigation required, there is enough evidence in the literature to begin to formulate some ideas about how PCa may arise from defective differentiation. In this section we will review our hypothesized model for how these pathways drive normal differentiation and summarize potential mechanisms for how their misregulation and crosstalk could alter Myc and Pten to drive oncogenesis.

### Model of normal differentiation

**Figure 2:**
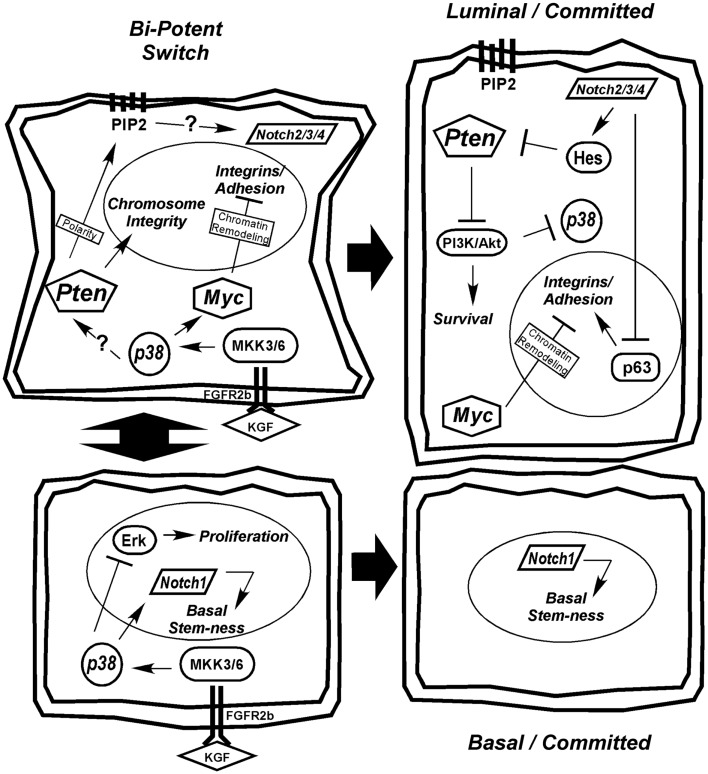
**Hypothetical model for prostate epithelial differentiation**. Upstream KGF(FGF7) from the stroma activates p38 through FGFR2b and MKK3/6, which plays multiple roles in differentiation, including inhibiting proliferation through suppression of Erk and upregulating differentiation determinants Notch1 and Myc. The transient-differentiating bipotent cell must make a decision between sustaining Notch1 signaling to commit to a basal phenotype or switching to Notch2/3/4 signaling to become luminal. The switch may be mediated by elevated Pten, via an unknown mechanism, to generate a PIP2 gradient at the apical membrane to establish cell polarity. Notch2/3/4 signaling in combination with Myc suppresses integrin expression allowing cells to detach. Luminal cells become dependent on PI3K signaling for survival as they lose matrix adhesion and integrin expression. Ultimately, Pten is partially suppressed to allow PI3K/Akt-dependent survival of the terminally differentiated luminal cell and the p38 differentiation program is terminated.

We favor the model of a transient-differentiating bipotent epithelial cell as the cell of origin for PCa. Based on the information presented in the preceding sections, we present a model of prostate epithelial differentiation (Figure 2). We hypothesize that the decision of a bipotent cell to differentiate toward a secretory phenotype is determined by stromal KGF (FGF7) activation of p38 through FGFR2b and MKK signaling. p38 affects a wide range of targets, including transcription factors, mRNA stability proteins, and other kinases/phosphatases. One action of p38 is to limit proliferation through Erk, while another is to upregulate Notch1 and Myc to drive differentiation. Upregulation of Pten, via a mechanism that may include Notch1 or other p38 target, is initially required to maintain chromosome stability and generate the PIP2 gradient at the apical membrane to establish cell polarity (Figure 2). The establishment of polarity is critical for initiating a differential gradient of Notch1 signaling basally to maintain stem-ness and Notch2/3/4 signaling apically toward the emerging luminal cell. Notch2/3/4 signaling in the emerging luminal cell downregulates p63, which along with Myc, downregulates integrin expression. Furthermore, as integrin expression diminishes in the transient-differentiating cell, there is a switch from Erk-dependent survival to a reliance on E-cadherin and PI3K survival pathways (17). Notch signaling through Hes1 limits Pten expression to allow effective PI3K/Akt signaling and survival of the luminal cell. Beyond promoting survival, PI3K negatively regulates Ask1, the upstream kinase required for p38 activation (272, 277, 294). Thus, PI3K upregulation in luminal cells may be required to terminate the p38 differentiation program (Figure 2).

**Figure 3:**
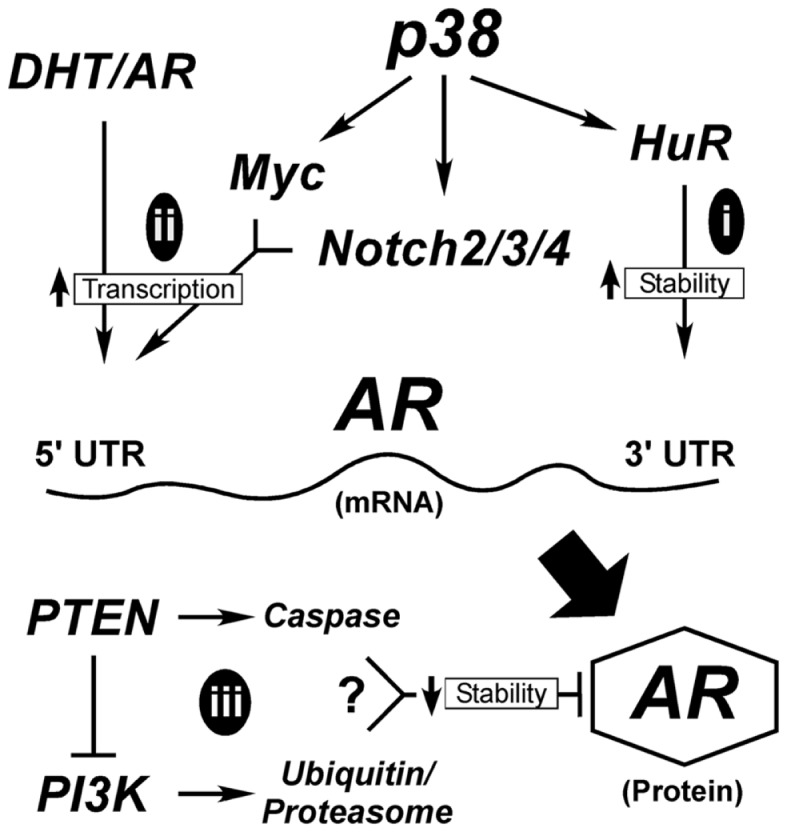
**Hypothetical mechanisms for AR upregulation during differentiation**. AR expression is tightly controlled both transcriptionally and post-transcriptionally. Early in differentiation prior to stable AR expression, p38 stimulates AR expression most likely by regulating mRNA stability **(i)**. Once low levels of AR are made, androgen (DHT) binding to AR stimulates its binding to its own promoter through AR binding elements (ARE). This requires the cooperation of signals from p38 that stimulate Myc and Notch2/3/4 to suppress cell adhesion, ultimately elevating AR mRNA transcription **(ii)**. AR protein is degraded through two different mechanisms **(iii)**, the classical ubiquitinylation system and through caspase 3. Both Pten and PI3K are reported to influence these degradation pathways; although how these two contradictory signals are balanced is not clear.

A critical question yet to be addressed in prostate secretory epithelial differentiation is how AR expression is upregulated. In Figure 3, we outline the few known mechanisms for controlling AR expression. Although, AR transcription is enhanced by androgen through AR and Myc binding sites in its promoter (Figure 3ii), this is not sufficient to induce AR expression in basal cells (17, 111). Low levels of AR mRNA or AR protein is detectable in AR-negative tumor cell lines (295, 296), suggesting AR expression is sensitive to both mRNA and protein stability factors. The mRNA PolyC-binding protein, HuR, binds polyC tracts in the 3′ UTR of AR mRNA and HuR (Figure 3i) is a known direct target of p38 (297–299). Thus, early signaling by p38 during differentiation may directly stabilize AR mRNA (Figure 3i). There are no reports linking Notch directly with AR expression, but stable AR protein expression requires loss of cell-matrix adhesion (17). Thus, the extent to which Notch is involved in reducing cell adhesion (300), would aid in enhancing AR expression.

Alternatively, AR protein stability may be enhanced during differentiation (Figure 3iii). Two mechanisms known to control AR protein stability are proteasomal degradation via ubiquitinylation and caspase 3-mediated degradation (301). Numerous AR-binding E3 ligases are known, but the upstream signals that suppress or activate them are less clear. One pathway involves Akt-dependent phosphorylation of Mdm2, which then targets AR for degradation (Figure 3). More intriguing is the role of Pten in promoting AR degradation by caspases (302). What is not clear is when during differentiation these pathways would be active, since they are antagonistic to each other and both result in AR loss. In Pten-negative tumors, active Akt would degrade AR resulting in AR loss. The reduced expression of AR in the prostate tumors of Pten-negative mice supports this idea (35). However, there must be a counteractive mechanism that keeps AR active in both tumors and secretory cells. Thus, even tumor cells are forced to balance signals to keep AR active.

### Mechanisms for oncogenic Myc upregulation

**Figure 4:**
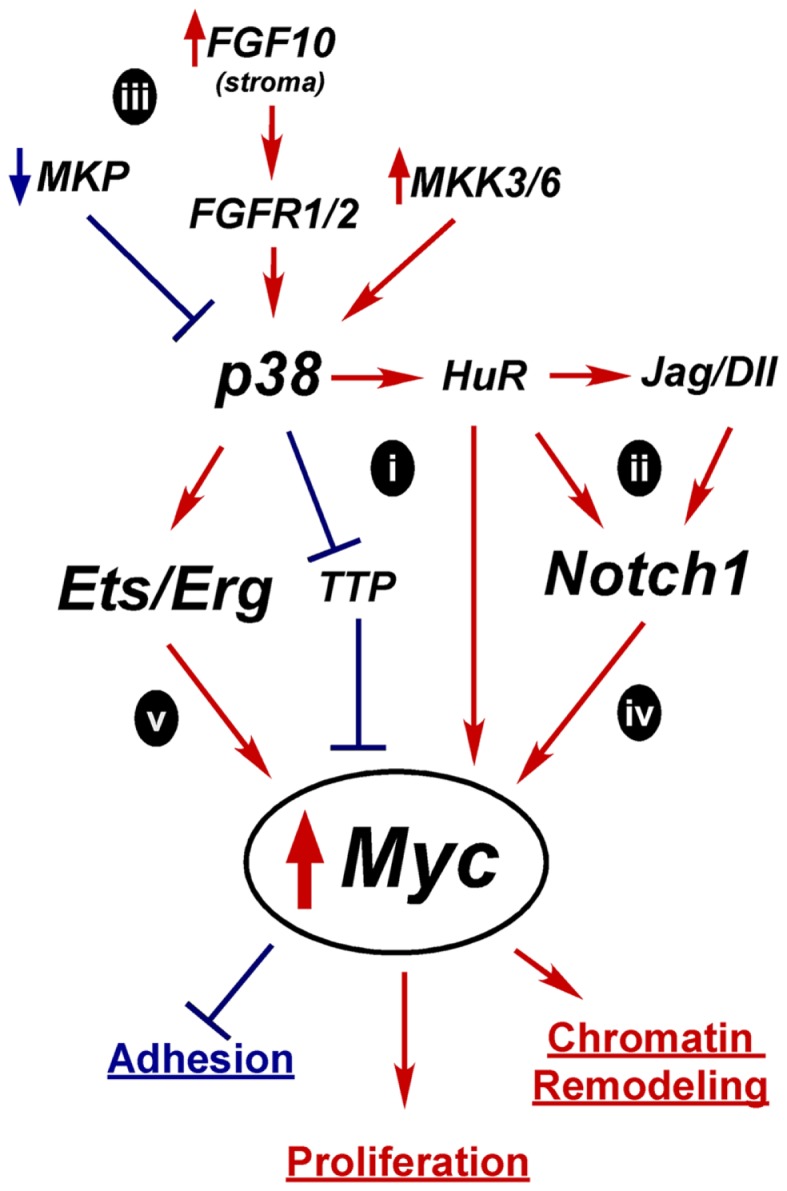
**Hypothetical mechanisms for Myc upregulation in prostate oncogenesis**. A tightly controlled increase in Myc expression is required for differentiation, but prolonged and excessive Myc activity is oncogenic and leads to increased proliferation, altered adhesion, and chromatin remodeling. Constitutive activation of p38 and Notch1 are two possible alternative mechanisms for upregulating Myc expression early in tumorigenesis. Active p38 can regulate mRNA stability proteins, such as TTP and HuR which affect Myc, Notch1, or Notch ligand mRNA half-life **(i,ii)**. Overactive p38 signaling could result from loss of MAPK phosphatases (MKP), alternate growth factor receptor expression, or activation of MKKs **(iii)**. Independently, overexpressed Notch ligands could stimulate Notch1 leading to increased Myc transcription **(iv)**. Activation of p38 may enhance Erg/Ets fusion gene activity to drive Myc overexpression**(v)**.

Genomic amplification of Myc is common in advanced tumors, but early tumors must have alternate mechanisms of Myc upregulation which are not well defined (51). A moderate but transiently sustained increase in Myc expression is required for differentiation, but something must happen to prolong Myc activation in oncogenesis (91). Both p38 and Notch are essential for differentiation and each can drive Myc expression. Thus, it is possible that misregulation of either could sustain Myc activation during tumorigenesis as depicted in Figure 4.

In the case of p38, one potential mechanism for increasing Myc activity is through mRNA stability (Figure 4i). Myc mRNA turnover is tightly controlled and p38 directly phosphorylates stability proteins that bind mRNAs like Myc that have stability control elements such as AU-rich or PolyC tracts (51, 303). p38 inactivates mRNA destabilizing proteins such as TTP, which upregulates Myc mRNA stability in rapamycin-treated cancer cells (304). Conversely, p38 activates HuR (297). HuR reportedly binds Myc mRNA, although there are conflicting reports as to whether this increases or decreases Myc stability (305–310). p38 may indirectly enhance Myc expression by increasing Notch ligand or receptor mRNA stability in an analogous fashion (Figure 4ii) (236). Notch1 has an AU-rich element and is stabilized via p38 activity in a leech zygote model (311). Additionally, the Notch ligand Dll4 was reported to be upregulated by HuR in mouse neuroepithelial cell (312). Whether Myc, Notch1, or Dll1 is regulated by p38 through mRNA stability in the prostate is unknown.

Several mechanisms could lead to aberrant p38 activation in tumors (Figure 4iii). Alterations that reportedly upregulate p38 signaling in PCa include loss of MAPK phosphatases, upregulation of upstream kinases MKK3/6, increased FGF10 expression in the stroma, and increased expression of proliferation-driving FGFRs (168). Prolonged p38 activation in a transiently differentiating bipotent cell would derail differentiation by preventing the required downregulation of Myc. Mutations that upregulate Notch1 signaling, such as increased ligand expression or increased receptor mRNA stability, could also lead to sustained Myc expression or activity (Figure 4iv).

The Ets fusion genes that are so prevalent in PCa provide another mechanism by which Myc could be upregulated (Figure 4v). TMPRSS2-Erg upregulates Myc in VCaP cells (77) and Etv4 binds Myc enhancers in PC3 cells (313). Etv4 knockdown decreases Myc expression. However, Etv4 overexpression in normal prostate epithelial cells did not alter Myc levels, suggesting other oncogenic alterations are required for this crosstalk (313). One of the downstream targets of p38 signaling is Elk-1, a member of the Ets family, which is upregulated in PCa (151). Thus, increased p38 signaling may be complementary or necessary for full Ets activation in PCa.

In summation, due to the normal upregulation of Myc as a part of differentiation, we hypothesize that alterations in one or more of these pathways would be enough to push Myc expression to initially stimulate differentiation. But its failure to subsequently be downregulated generates an oncogenic event resulting in only a partially committed, yet proliferative, cell with an altered chromatin program and retained matrix adherence. This model will need further testing to determine its relevance in human prostate epithelial differentiation and oncogenesis.

### Mechanisms for oncogenic Pten downregulation

**Figure 5:**
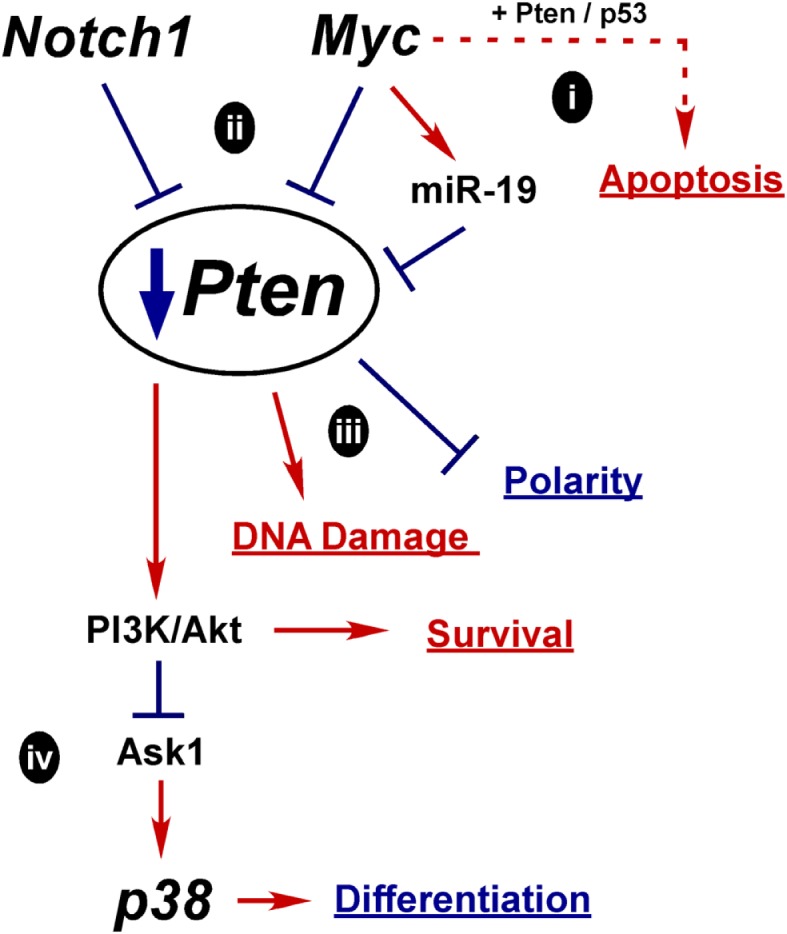
**Hypothetical mechanisms for Pten down regulation in prostate oncogenesis**. Myc or Notch1 could each act to suppress Pten expression early in oncogenesis. Loss of Pten may be a prerequisite for Myc upregulation to relieve Myc induced apoptosis **(i)**. However, moderate activation of Myc could also contribute to Pten downregulation, via either transcriptional repression or induction of miR-19. Increased Notch1 signaling may also transcriptionally downregulate Pten **(ii)**. Ultimely downregulation of Pten can lead to more DNA damage, loss of polarity, and increased PI3K/Akt survival signaling **(iii)**. Loss of Pten can lead to stimulation of p38 via activation of upstream MAPKs, such as Ask1, further contributing to oncogenesis**(iv)**.

Another key oncogenic event in PCa is downregulation of Pten. There are several potential mechanisms whereby misregulation of normal differentiation signals could lead to Pten loss as outlined in Figure 5. How Pten expression is controlled during differentiation is not clear, but it is required to maintain epithelial integrity and structure. In fact, downregulation of Pten may be a prerequisite to Myc-driven oncogenesis, since elevated and prolonged Myc expression can induce apoptosis, potentially via Pten activation of p53 and Rad51 (Figure 5i) (58, 95, 314).

About 30–40% of tumors with loss of Pten protein do not show genomic loss of the gene, indicating alternate mechanisms of inactivation (60). Crosstalk between Pten, Notch, and Myc is reported in the literature and we hypothesize that oncogenic action of Notch or Myc is sufficient to limit Pten expression early in oncogenesis (Figure 5ii). One mechanism may be similar to what is observed in Notch-driven T-ALL, where Hes1 and Myc both transcriptionally repress Pten (315, 316). In T-ALL, Notch inhibition kills cells by blocking Pten downregulation; however, resistant cells develop alternate mechanisms to upregulate Akt (316). In the prostate, NICD1 expression in mouse luminal cells (via the probasin promoter) leads to hyperplasia and decreases Pten expression in both epithelial and stromal cells (225). Likewise, CSL knockout in luminal cells (via the Nkx3.1 promoter) leads to increased Pten. Additionally, Myc activation of miR-19 targets and downregulates Pten in a mouse Myc-driven B-cell lymphoma model (317). Thus, under oncogenic conditions, upregulated Notch or Myc suppresses Pten expression and potentially drives tumor initiation (Figure 5ii) (223, 317).

We further hypothesize that Pten aids in temporally or spatially controlling the Notch switch that commits the cell to either the basal (Notch1) or luminal (Notch2/3/4) lineage (Figure 2). The necessary oncogenic consequence of Pten inactivation would be disruption of cell polarity, failure to maintain epithelial structure, and impaired differentiation (Figure 5iii). Because Pten is also critical for controlling DNA damage, another consequence of Pten loss would be increased susceptibility of the cell to mutation.

The best characterized result of Pten loss is increased survival signaling through PI3K/Akt, which is consistent with the switch to dependence on PI3K/Akt for normal secretory cell survival during differentiation (17). Another consequence of PI3K/Akt activation is suppression Ask1 (Figure 5iv), the upstream kinase required for p38 activation (272, 277, 294). If suppression of p38 signaling due to Pten loss occurs too early in the differentiation program, cells will not complete the process and become arrested. However, PI3K activation in tumor cells was shown to activate p38, which then stimulates EGFR and creates a positive feedback loop further activating PI3K (318). The mechanism of how PI3K activates p38 is not clear, but other oncogenic mutations may reverse the normal regulation by Ask1 and activate p38. Whether any of these events are major mechanisms for Pten loss or whether the downstream consequences are critical for prostate oncogenesis has yet to be demonstrated, but this model provides a sampling of potential mechanisms that should be further investigated.

## Conclusion

In conclusion, pathways known to be involved in normal prostate differentiation (Myc, p38, Notch, and PI3K/Pten) can be linked to PCa. These pathways have complicated interactions; multiple functions, multiple targets, many levels of regulation, and significant crosstalk. To better understand prostate oncogenesis, these pathways need to be more thoroughly investigated in human prostate models. Differentiation is a very tightly regulated process where cells undergo major reprograming. We have provided a few, though certainly not exhaustive, potential models for how oncogenic disruption of specific differentiation pathways can drive tumor initiation. We hypothesize that the oncogenic cell of origin for PCa is not a committed basal or luminal stem cell, but rather a transient-differentiating bipotent cell. The bipotent K5/K8 cells found in both human and mouse prostates are the most likely candidates for prostate oncogenic transformation (25, 32). These cells must maintain very tight temporal and spatial control of the differentiation pathways and thus may be the easiest targets for oncogenic disruption. Only through better understanding of oncogenesis will we find new ways to classify prostate tumors and better predict tumor aggressiveness. Such discoveries will ultimately provide physicians and patients with more information when deciding how to treat this widespread disease.

## Authors Contribution

Sander B. Frank was responsible for researching and writing the review. Cindy K. Miranti was responsible for editing, validating citations, and communicating the review.

## Conflict of Interest Statement

The authors declare that the research was conducted in the absence of any commercial or financial relationships that could be construed as a potential conflict of interest.
